# Significant genomic introgression from grey junglefowl (*Gallus sonneratii*) to domestic chickens (*Gallus gallus domesticus*)

**DOI:** 10.1186/s40104-024-01006-7

**Published:** 2024-04-01

**Authors:** Xiurong Zhao, Junhui Wen, Xinye Zhang, Jinxin Zhang, Tao Zhu, Huie Wang, Weifang Yang, Guomin Cao, Wenjie Xiong, Yong Liu, Changqing Qu, Zhonghua Ning, Lujiang Qu

**Affiliations:** 1 National Engineering Laboratory for Animal Breeding, College of Animal Science and Technology, China Agricultural University, Beijing, 100193 China; 2 Key Laboratory of Protection and Utilization of Biological Resources in Tarim Basin, Xinjiang Production and Construction Corps, Tarim University, Alar, 843300 China; 3 Beijing Municipal General Station of Animal Science, Beijing, 100107 China; 4 Animal husbandry station of Fangchenggang, Fangchenggang, Guangxi Province 538001 China; 5 Animal Disease Prevention and Control Center of Fangchenggang, Fangchenggang, Guangxi Province 538001 China; 6 Beijing Agricultural Effect Poultry Industry Co., Ltd., Beijing, 101100 China; 7 Engineering Technology Research Center of Anti-aging Chinese Herbal Medicine of Anhui Province, Fuyang Normal University, Fuyang, Anhui 236037 China

**Keywords:** *BCO2*, Domestic chickens, Grey junglefowl, Introgression

## Abstract

**Background:**

Chicken is one of the most numerous and widely distributed species around the world, and many studies support the multiple ancestral origins of domestic chickens. The research regarding the yellow skin phenotype in domestic chickens (regulated by *BCO2*) likely originating from the grey junglefowl serves as crucial evidence for demonstrating the multiple origins of chickens. However, beyond the *BCO2* gene region, much remains unknown about the introgression from the grey junglefowl into domestic chickens. Therefore, in this study, based on whole-genome data of 149 samples including 4 species of wild junglefowls and 13 local domestic chicken breeds, we explored the introgression events from the grey junglefowl to domestic chickens.

**Results:**

We successfully detected introgression regions besides *BCO2*, including two associated with growth trait (*IGFBP2* and *TKT*), one associated with angiogenesis (*TIMP3*) and two members of the heat shock protein family (*HSPB2* and *CRYAB*). Our findings suggest that the introgression from the grey junglefowl may impact the growth performance of chickens. Furthermore, we revealed introgression events from grey junglefowl at the *BCO2* region in multiple domestic chicken breeds, indicating a phenomenon where the yellow skin phenotype likely underwent strong selection and was retained. Additionally, our haplotype analysis shed light on *BCO2* introgression event from different sources of grey junglefowl into domestic chickens, possibly suggesting multiple genetic flows between the grey junglefowl and domestic chickens.

**Conclusions:**

In summary, our findings provide evidences of the grey junglefowl contributing to the genetic diversity of domestic chickens, laying the foundation for a deeper understanding of the genetic composition within domestic chickens, and offering new perspectives on the impact of introgression on domestic chickens.

**Supplementary Information:**

The online version contains supplementary material available at 10.1186/s40104-024-01006-7.

## Background

As an essential poultry species closely associated with the origin and development of human agricultural civilization, chicken has been raised worldwide [1]. They are not only used for meat and egg production but also for ornamental and recreational purposes. And they have become the most numerous animals among livestock. Domestic chickens belong to the genus *Gallus*, which includes 4 diverse wild junglefowl species, the green junglefowl (*G. varius*), in Java and surrounding islands in Indonesia, the Ceylon junglefowl (*G. lafayettii*) in Sri Lanka, the grey junglefowl (*G. sonneratii*), native to Southern and Western India, and the red junglefowl (*G. gallus*), widely distributed across South Asia and Southeast Asia [2]. The red junglefowl further consists of 5 subspecies: *Gallus gallus bankiva* (*G. g. bankiva*) in Java, *Gallus gallus murghi* (*G. g. murghi*) in India, Kashmir, Nepal, Bangladesh, and Bhutan, *Gallus gallus jabouillei* (*G. g. jabouillei*) in North Vietnam and South China, *Gallus gallus gallus* (*G. g. gallus*) in Southeast Asia mainland (Thailand, Laos, Vietnam, and Cambodia), and *Gallus gallus spadiceus* (*G. g. spadiceus*) in Southwest China, Peninsular Malaysia, India, and Myanmar [1].

However, the issue of the domestic chickens’ origin has long been a contentious topic. Initially, Charles Darwin proposed that the red junglefowl was the ancestor of domestic chickens [3], a widely embraced viewpoint. With the advancement of molecular biology techniques, this perspective began to be challenged. Some researchers conducted haplotype analysis based on mitochondrial DNA sequences of domestic chickens and wild junglefowl species, which pointed toward a polyphyletic origin for domestic chickens, implying that domestic chickens may have genetic lineage from the other wild junglefowl species [4]. Later, upon the publication of the domestic chicken whole-genome sequence, Eriksson et al. used the yellow skin phenotype of domestic chickens as a point of entry, based on phylogenetic analysis, they further proposed that the yellow skin phenotype in domestic chickens likely originated from the grey junglefowl [5]. This study provides the first evidence suggesting the potential introduction of genetic lineage from the other wild junglefowl species during the domestication of chickens. Subsequently, studies by Wang et al. [6] and Lawal et al. [7]  furtherly revealed that the red junglefowl is the primary wild ancestor of domestic chickens, and the other three junglefowl species also contribute to the genetic diversity of domestic chickens.

Actually, interspecies introgression and gene flow are frequently observed in the domestication of animals [8], serving as a crucial mechanism for introducing novel genetic variations [9]. Currently, in a variety of animals and plants, instances of introgression between wild and domesticated species are gradually being reported. Examples include introgression from markhor to goats [9], wild relatives to domestic sheep populations (argali to Bashibai, Asiatic mouflon to Grey Shiraz, European mouflon to Caucasian [10], argali to Tibetan sheep [11, 12]), European wild grapes to cultivated wine grapes[13], wild emmer to modern wheat [14] and so on.

Presently, the introgression event from the wild junglefowl to the genome of domestic chickens is still relatively limited. Regarding the introgression from the grey junglefowl into domestic chickens, the significant study is by Eriksson et al. [5], who demonstrated that the *BCO2* (beta-carotene oxygenase 2) region, which regulates yellow skin phenotype, likely originated from the grey junglefowl. Lawal et al. further detected genomic regions introgressed by the grey junglefowl in local chicken populations from South Asia, Central Asia, and Africa [7]. However, whether domestic chickens from the other regions have experienced introgression and to what extent remains unexplored. Wang et al. [6] conducted a study using domestic chickens from various regions worldwide, further confirming the contribution of the grey junglefowl to the genetic diversity of domestic chickens. Still, they did not delve into detailed exploration of introgressed gene regions. In summary, there are many unknown introgression regions that require further investigation and exploration.

Thus, in the present study, we analyzed the genome sequence of 149 chickens including green junglefowl, grey junglefowl, Ceylon junglefowl, red junglefowl, and 13 domestic chicken breeds. We utilized genomic data to investigate the introgression from the grey junglefowl into domestic chickens, further exploring the introgression regions beyond the *BCO2* gene region in the chicken genome. And finally, we identified that the introgression from the grey junglefowl could potentially impact the growth performance of domestic chickens. Additionally, we furtherly uncovered evidence of multiple genetic flows between the grey junglefowl and domestic chickens. Our findings offer novel insights into comprehending the genetic diversity and origins of domestic chickens.

## Methods

### Samples and sequencing

A total of 149 samples were used in this study, of which including 15 green junglefowl, 9 grey junglefowl, 10 Ceylon junglefowl, 16 red junglefowl, 10 Canada indigenous chickens, 4 India local chickens and 11 Chinese indigenous chicken breeds. The 11 Chinese chicken breeds comprise Beijing You chickens, Heilongjiang Lindian chickens, Jiangsu Liyang chickens, Hubei Yunyang Da chickens, Guangdong Xinghua chickens, Guangxi Dongzhong Dwarf chickens, Hainan Wenchang chickens, Xinjiang Hetian local chickens, Jiangxi local chickens, Shandong local chickens, and Taiwan local chickens. Among the samples used, 25 samples were sequenced for this study, while the remaining were downloaded from public databases (http://bigd.big.ac.cn/chickensd [6], DRA003951 [15], PRJNA432200 [7], PRJNA552030 [16], PRJNA720223 [17], SAMN14651083 [18], PRJNA800119 [19] ). The detail information of samples could be found in the Additional file 1: Table S1. For the samples generated in this study, we extracted the genomic DNA using the routine phenol-chloroform method and performed sequencing on Illumina HiSeq 2500 system.

### Quality control and variant calling

Raw reads were filtered by fastp [20]. And then, the filtered clean reads were mapped to the reference genome (Gallus gallus 6.0) using the Burrows-Wheeler Aligner (BWA) software [21] with default parameters. The aligned bam files were sorted using the Samtools (v.1.11) [22], and duplicated reads were marked with the “MarkDuplicates” module of Picard (https://broadinstitute.github.io/picard). Next, we performed SNP calling with the “Haplotypecaller” and “GenotypeGVCFs” parameter of the GATK [23]. Variants with “QUAL < 30.0 || QD < 5.0 || FS > 60.0 || MQ < 40.0 || MQRankSum < −12.5 || ReadPosRankSum < −8.0” were filtered using “VariantFiltration” function of GATK. Finally, 15,371,798 SNPS were obtained for the subsequent analysis.

### Population structure analysis

To evaluate the population structure, we performed PCA (principal component analysis) using PLINK [24]. And then, we constructed a maximum likelihood (ML) tree implemented in SNPhylo [25] to reveal phylogenetic relationship. The ML tree was furtherly visualized using iTOL (https://itol.embl.de). Additionally, we used the Admixture software [26] to estimate the genetic composition of the populations by assuming the ancestral clusters (K) from 2 to 15. The final genetic structure and cluster results were visualized using Pophelper (http://pophelper.com) [27]. To mitigate the impact of LD on the population structure, we employed SNPs pruning with the "--indep-pairwise 50 5 0.05" option in PLINK.

### Genomic introgression analysis

*D*-statistic method was employed in this study to detect introgression from the grey junglefowl to domestic chickens. *D*-statistic is also known as the ABBA-BABA test. Given a tree topology (((P1, P2), P3), O), the ABBA event indicates P2 shares more derived alleles with P3 (there are gene flow between P2 and P3), the BABA pattern indicates P1 shares more derived alleles with P3 (there are gene flow between P1 and P3). In our study, we used the green junglefowl as an outgroup, P1 is red junglefowl, P2 is a domestic chicken population, P3 is grey junglefowl. We performed *D*-statistic with Dsuite software [28]. First, we used Dtrios module to calculate *D* and *f*_4_-ratio statistics for all possible trios of populations. Subsequently, we used Dinvestigate module to evaluate the introgression level and locate the introgression region across the whole genome, using a sliding window contained 100 informative SNPs with a step of 20 SNPs. We considered the windows in the top 1% of the distribution of *D* values as the candidate introgression region. Then, we annotated genes in the related introgression region with the Biomart module of Ensembl (http://www.ensembl.org/biomart/martview).

Subsequently, we constructed the ML tree for the introgression genomic region using IQ-TREE [29] and visualized with online iTOL. And we compared the mean pairwise sequence divergence (dxy) between the introgressed domestic chickens and the grey junglefowl/the other domestic chicken using a 50-kb sliding window and a stepping of 10-kb [30].

In addition, to further examine the introgression event of yellow skin in domestic chickens, we constructed a haplotype network for *BCO2* region using the POPART software [31] with default parameters.

## Results

### Population structure

**Fig. 1:**
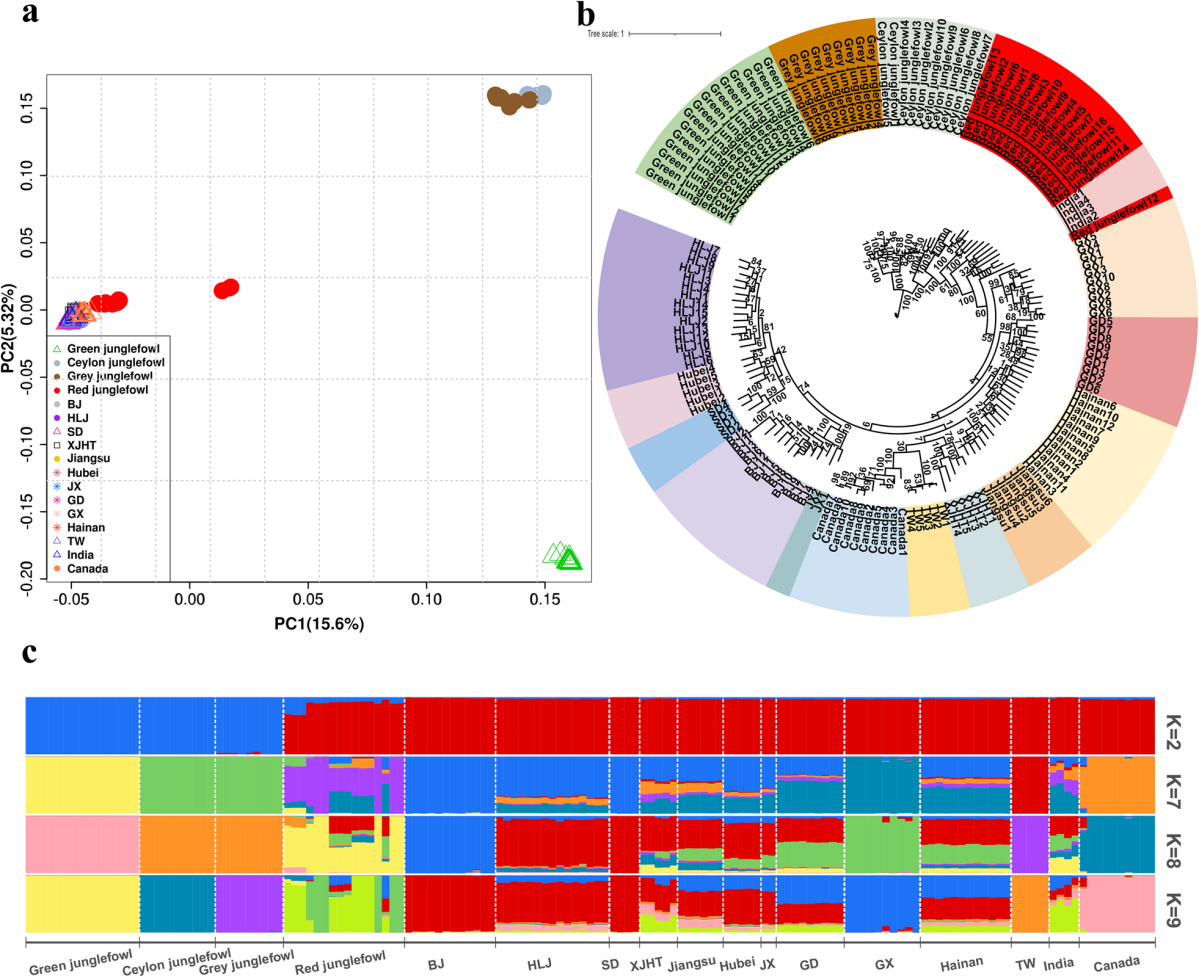
Population structure analysis of samples in this study. **a**. PCA plot of samples in this study. The abbreviation of domestic chickens could be found in Additional file 1: Table S1. **b**. Phylogenetic tree of the samples in this study. **c**. Admixture results for K = 2, K = 7, K = 8 and K = 9

One hundred and forty-nine samples including 4 species of wild junglefowls and 13 local domestic chicken breeds were analyzed in the present study (Additional file 1: Table S1). After aligning the reads to the reference genome, 18.51 billion mapped reads were obtained, with an average sequencing depth of 15.5X per individual (Additional file 1: Table S2). Following variation calling and quality filtration, a total of 15.37 million SNPs were obtained for the subsequent analysis. First, we performed PCA to elucidate the genetic relationship among the populations in this study. In our PCA result, PC1 (the first principal component) could clearly separates 3 wild junglefowl species (green junglefowl, Ceylon junglefowl, and grey junglefowl) from domestic chickens, with domestic chickens exhibit a closer genetic clustering with the red junglefowl. Furthermore, we observed that the Ceylon junglefowl and grey junglefowl cluster together (Fig. 1a). Subsequently, a ML phylogenetic tree was constructed. The results show that domestic chickens could cluster according to the geographic origins and are closely clustered with the red junglefowl. And the Ceylon junglefowl and grey junglefowl were observed to cluster together (Fig. 1b). Additionally, we used Admixture to infer the population admixture proportions by assuming K from 2 to 15. When K = 2–8, we observed that the Ceylon junglefowl and grey junglefowl share common ancestral components, indicating a close relationship between these two junglefowl species. At K = 9, we found the separation of the Ceylon junglefowl and grey junglefowl into distinct clusters (Fig. 1c). In conclusion, all the aforementioned results support the sister-group relationship between the Ceylon junglefowl and grey junglefowl, as well as a closer relationship between the red junglefowl and domestic chickens.

### Introgression analysis

**Fig. 2:**
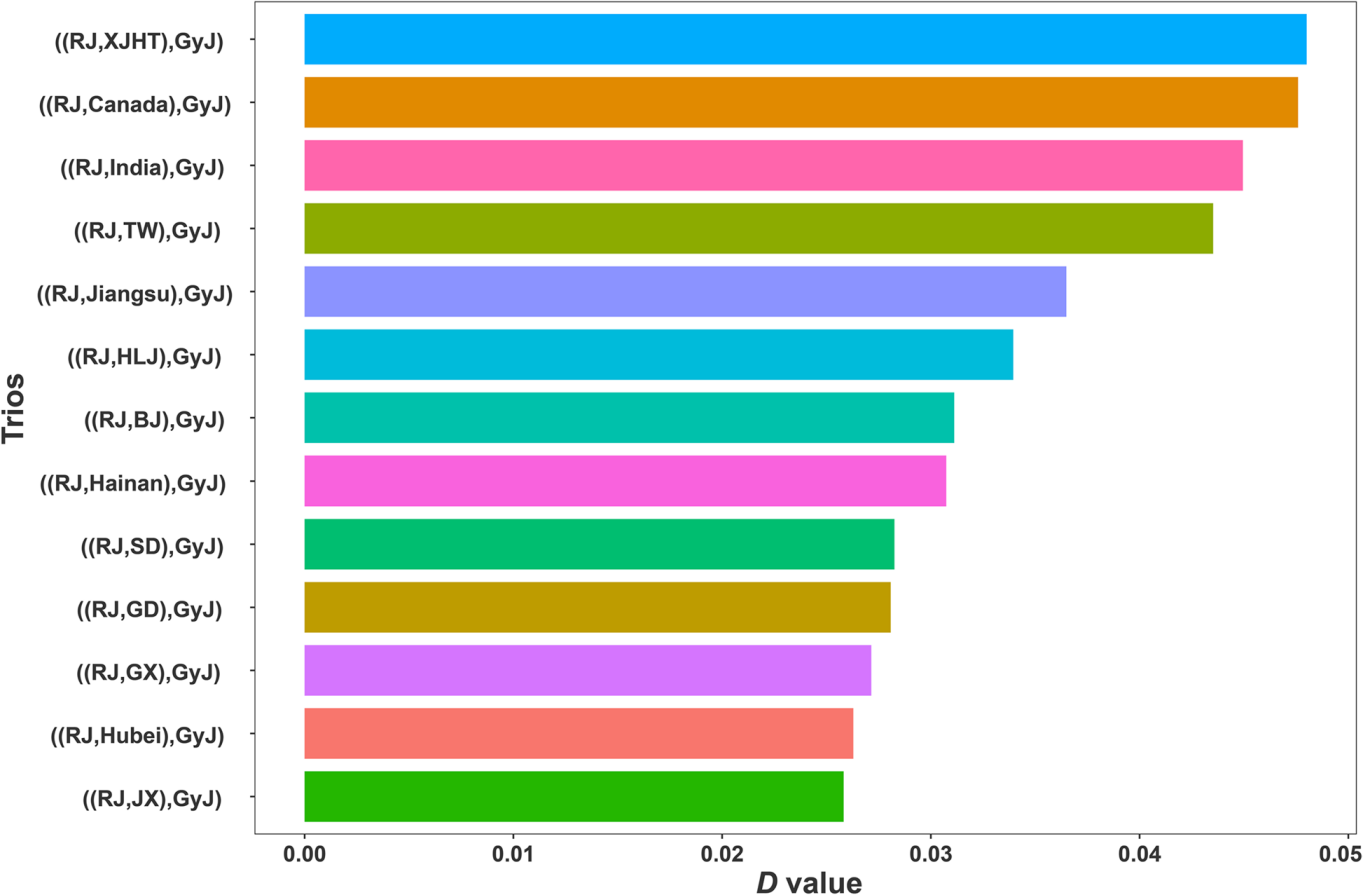
The result of *D*-statistics for introgression between the grey junglefowl and domestic chickens. The RJ is red junglefowl, the GyJ is grey junglefowl, and the remaining abbreviation of domestic chickens could be found in Additional file 1: Table S1

To investigate the introgression events from the grey junglefowl to domestic chickens, we calculated the *D*-statistics for each combination of domestic chicken populations and grey junglefowl. The result of *D*-statistics showed that all domestic chicken populations in this study exhibited significant introgression (Z > 3, *P* < 0.001), and the fraction of introgression ranging from 1.28% to 2.43% (Fig. 2 and Additional file 1: Table S3). We identified ((RJ, XJHT), GyJ) and ((RJ, Canada), GyJ) having the most significant *D* value and *f*_4_-ratio, indicating a relative high introgression level from the grey junglefowl to XJHT and Canada indigenous chickens, at 2.39% and 2.43%, respectively. Consequently, following that, we separately studied the genomic regions shared between the grey junglefowl and local chickens from XJHT, as well as between the grey junglefowl and chickens from Canada.

### Introgression from grey junglefowl to XJHT indigenous chickens

**Fig. 3:**
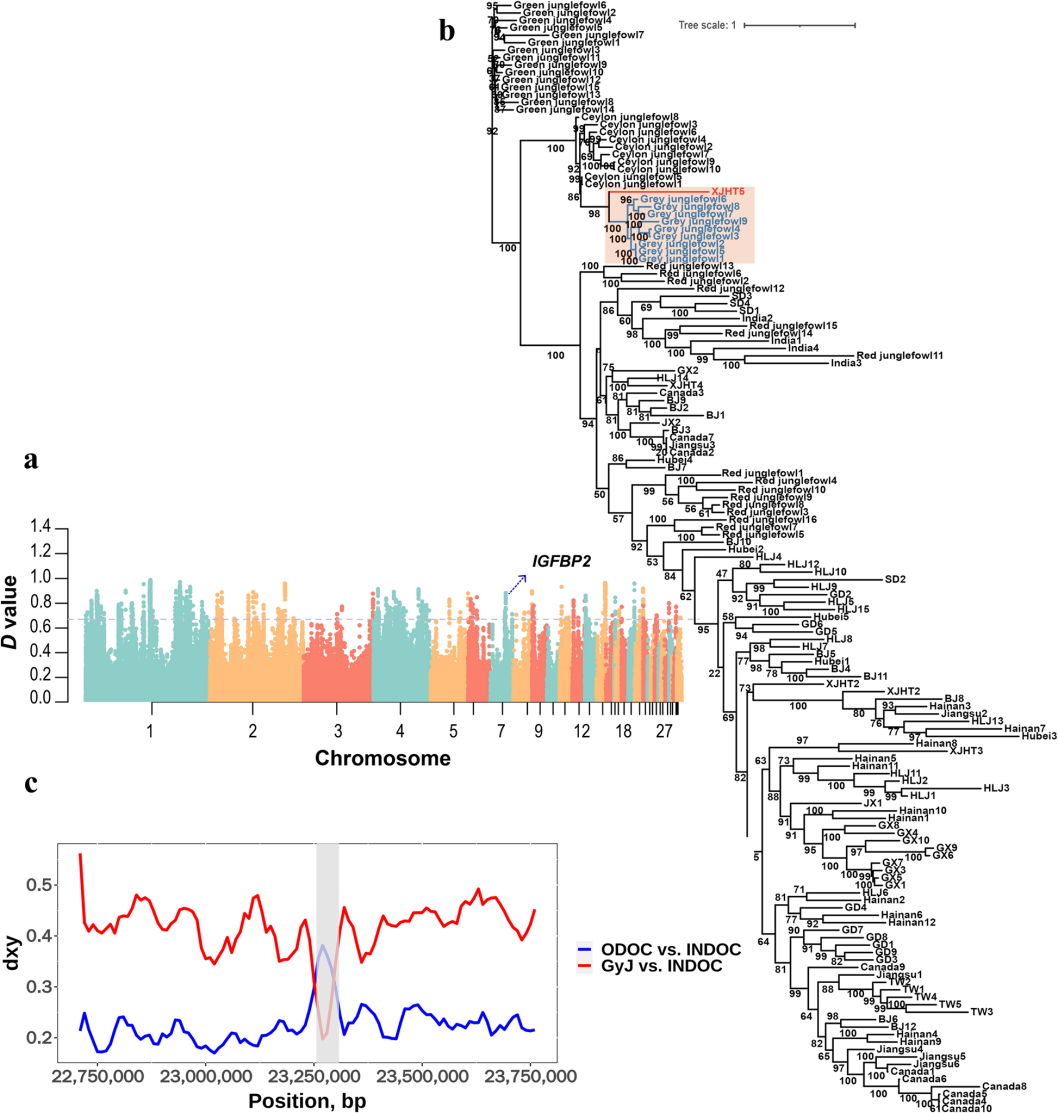
Introgression at the *IGFBP2* gene region. **a**. Manhattan plot of *D* values between XJHT local chickens and grey junglefowl. The dashed line indicates the significance threshold (top 1% of the distribution of *D* values). **b**. ML tree constructed with the *IGFBP2* gene sequence. The introgression event was highlighted with orange color, the introgressed XJHT individual and the grey junglefowl were highlighted with red and blue color, respectively. **c**. Mean pairwise sequence divergence at the *IGFBP2* gene region between the introgressed XJHT local chickens (INDOC) and either grey junglefowl (GyJ) or remaining non-introgressed XJHT local chickens (ODOC), represents by red and blue lines, respectively. The shaded area represents the *IGFBP2* gene region

**Fig. 4:**
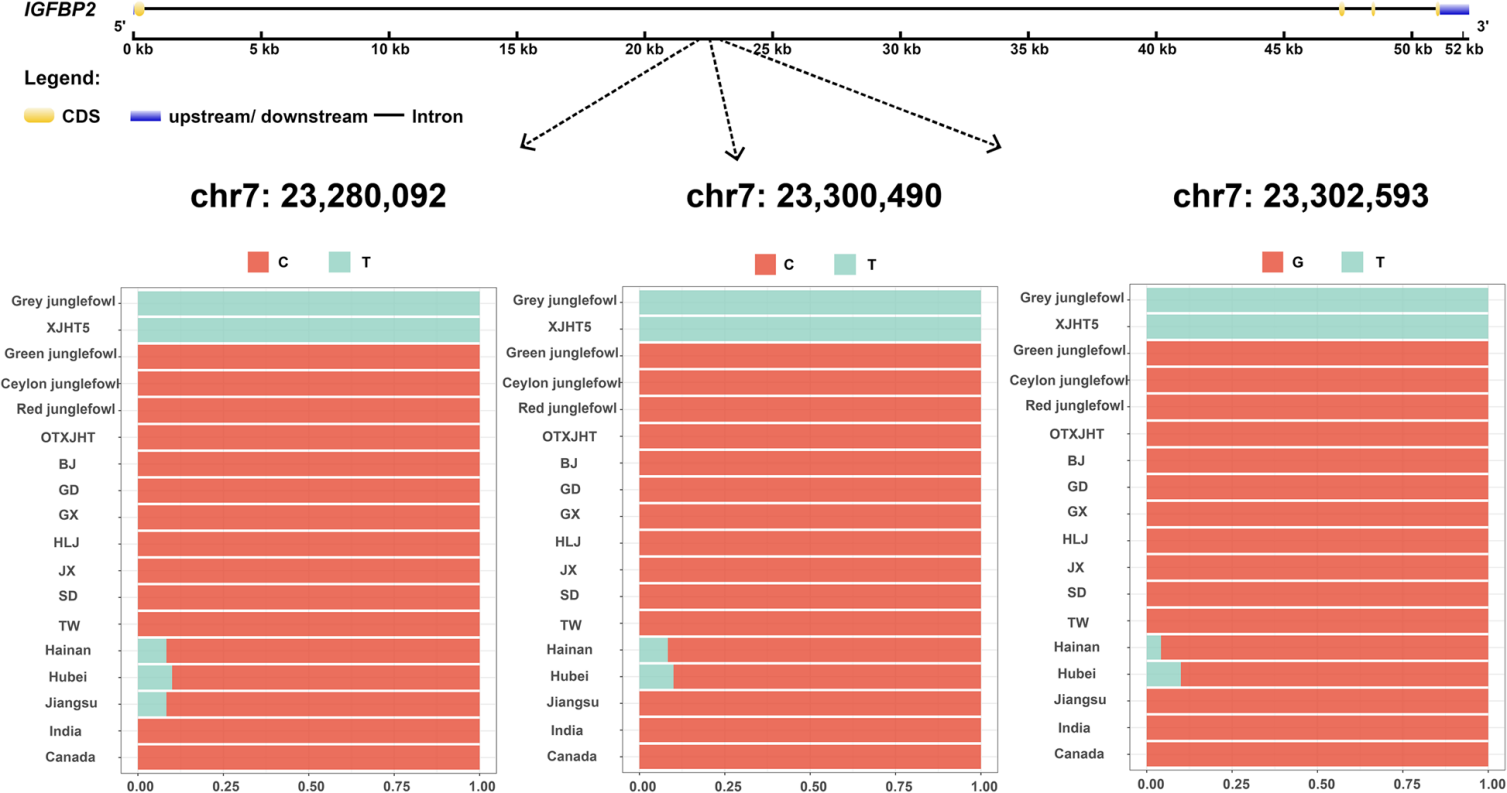
Allele frequency distribution of 3 SNPs (chr7: 23,280,092, chr7: 23,300,490 and chr7: 23,302,593) within *IGFBP2* in the wild junglefowl, introgressed XJHT individual (XJHT5), non-introgressed XJHT chickens (OTXJHT), and the remaining domestic chickens

We further detected 173 candidate introgression genes from the grey junglefowl to XJHT indigenous chickens (Fig. 3a and Additional file 1: Table S4). In particular, we identified two genes related to the development of growth and angiogenesis including *IGFBP2* (insulin like growth factor binding protein 2) and *TIMP3* (TIMP metallopeptidase inhibitor 3). Taking *IGFBP2* as an example, we first constructed an ML tree for this gene region. In the ML tree, we noticed that XJHT5 clustered with the grey junglefowl, while the remaining XJHT local chickens clustered together with the other domestic chickens (Fig. 3b and Table 1). Moreover, for this region, we observed a remarkably reduced dxy between XJHT5 and the grey junglefowl, in contrast to the significantly increased dxy in XJHT5 versus the other XJHT local chickens (Fig. 3c). To further understand the introgression event in the *IGFBP2* region, we conducted a study on the gene variation. We found 3 SNPs were exhibiting homozygous mutations in both grey junglefowl and the introgressed XJHT5 individual, while these mutations were either absent or have extremely low allele frequencies in the other junglefowl and domestic chickens (Fig. 4). The result reconfirmed the introgression event at the *IGFBP2* gene region. Additionally, we also performed ML tree construction and dxy comparison in the *TIMP3* related to angiogenesis. The result was consistent with those above, and further supported the introgression from the grey junglefowl to XJHT chickens (Additional file 2: Fig. S1 and Table 1).

**Table 1:** The detail information of 6 introgressed genes

Gene name	Chromosomal position	Breeds shown introgression	Number of introgressed animals	IDs of introgressed animals
*TIMP3*	1:53,094,947–53,127,580	XJHT	1	XJHT4
*IGFBP2*	7:23,255,548–23,307,791	XJHT	1	XJHT5
*TKT*	12:7,348,689–7,371,332	Canada	4	Canada2, Canada3, Canada8, Canada9
*BCO2*	24:6,140,068–6,160,788	Hainan, GX, BJ, Canada, HLJ, GD, Jiangsu	74	Hainan1, Hainan2, Hainan3, Hainan4, Hainan5, Hainan6, Hainan7, Hainan8, Hainan9, Hainan10, Hainan11, Hainan12, GX1, GX2, GX3, GX4, GX5, GX6, GX7, GX8, GX9, GX10, BJ1, BJ2, BJ3, BJ4, BJ5, BJ6, BJ7, BJ8, BJ9, BJ10, BJ11, BJ12, Canada1, Canada2, Canada3, Canada4, Canada5, Canada6, Canada7, Canada8, Canada9, Canada10, HLJ1, HLJ2, HLJ3, HLJ4, HLJ5, HLJ6, HLJ7, HLJ8, HLJ9, HLJ10, HLJ11, HLJ12, HLJ13, HLJ14, HLJ15, GD1, GD2, GD3, GD4, GD5, GD6, GD7, GD8, GD9, Jiangsu1, Jiangsu2, Jiangsu3, Jiangsu4, Jiangsu5, Jiangsu6
*HSPB2*	24:6,227,008–6,259,713	Hainan, GX, SD, BJ, Canada, HLJ	14	Hainan6, Hainan10, GX1, GX9, GX10, BJ6, BJ11, SD1, Canada4, Canada9, Canada10, HLJ2, HLJ4, HLJ6
*CRYAB*	24:6,239,995–6,246,232	Hainan, GX, SD, BJ, Canada, HLJ	14	Hainan6, Hainan10, GX1, GX9, GX10, BJ6, BJ11, SD1, Canada4, Canada9, Canada10, HLJ2, HLJ4, HLJ6

### Introgression from grey junglefowl to Canada indigenous chickens

**Fig. 5:**
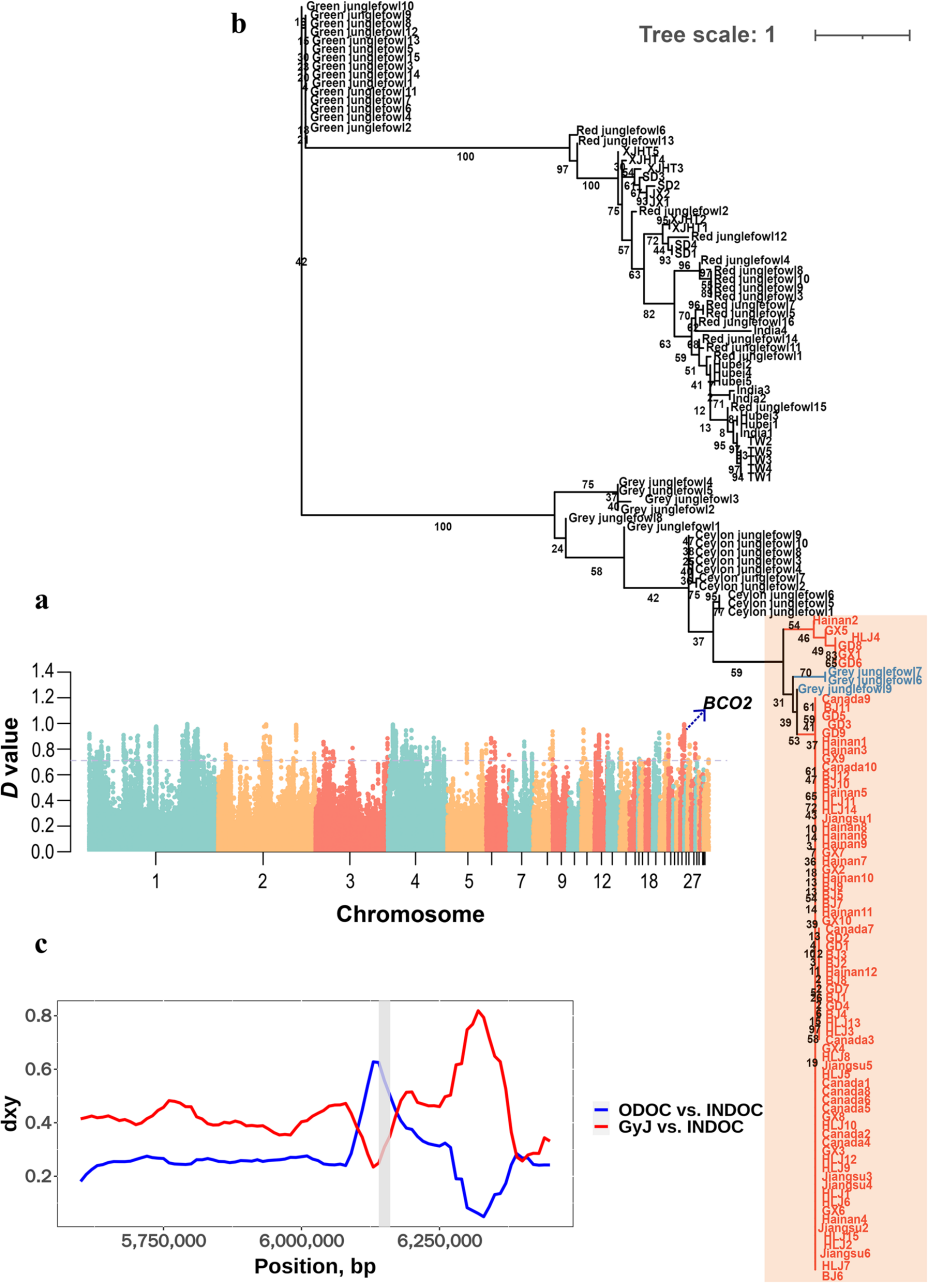
Introgression at the *BCO2* gene region. **a**. Manhattan plot of *D* values between Canada local chickens and grey junglefowl. The dashed line indicates the significance threshold (top 1% of the distribution of *D* values). **b**. ML tree constructed with the *BCO2* gene sequence. The introgression event was highlighted with orange color, the introgressed chickens and the grey junglefowl were highlighted with red and blue color, respectively. **c**. Mean pairwise sequence divergence at the *BCO2* gene region between the introgressed individuals (INDOC) and either grey junglefowl (GyJ) or remaining non-introgressed domestic chickens (ODOC), represents by red and blue lines, respectively. The shaded area represents the *BCO2* gene region

In Canada local chickens, a 20 kb region (within the range of 6.14–6.16 Mb) on chromosome 24 exhibited a significant signal of admixture with the grey junglefowl (Fig. 5a and Additional file 1: Table S5). This region contains *BCO2* gene, which regulates the yellow skin phenotype. We applied the same methods to study the *BCO2* gene as mentioned for *IGFBP2*. In the ML tree, we found that some domestic chickens clustered together with the grey junglefowl, while the rest of the domestic chickens formed a separate cluster with the red junglefowl (Fig. 5b and Table 1), consistent with the previous studies [7]. Notably, the chickens clustering with the grey junglefowl included not only Canada indigenous chickens but also those from BJ, GD, GX, HLJ, Hainan, and Jiangsu. To validate the results observed from the phylogenetic tree, we further examined the regions originating from the grey junglefowl in the genomes of these domestic chicken populations (Additional file 1: Table S6–S11). The results confirmed that *BCO2* was commonly identified as a candidate introgression region in these populations, supporting the findings from the ML tree. Furthermore, the result of dxy indicated that within this region, the dxy between the introgressed individuals and the grey junglefowl was significantly decreased, while the dxy between the introgressed individuals and the other domestic chickens was significantly increased (Fig. 5c). Additionally, we also identified one gene related to growth regulation (*TKT* (transketolase)) and two heat shock protein members (*CRYAB* (crystallin alpha B), *HSPB2* (heat shock protein family B)). ML tree and dxy results of these gene regions all indicate admixture between the grey junglefowl and Canada indigenous chickens (detail see Table 1, Additional file 3: Fig. S2 and Additional file 4: Fig. S3).

### Exploring the origin of yellow skin phenotype in domestic chickens

**Fig. 6:**
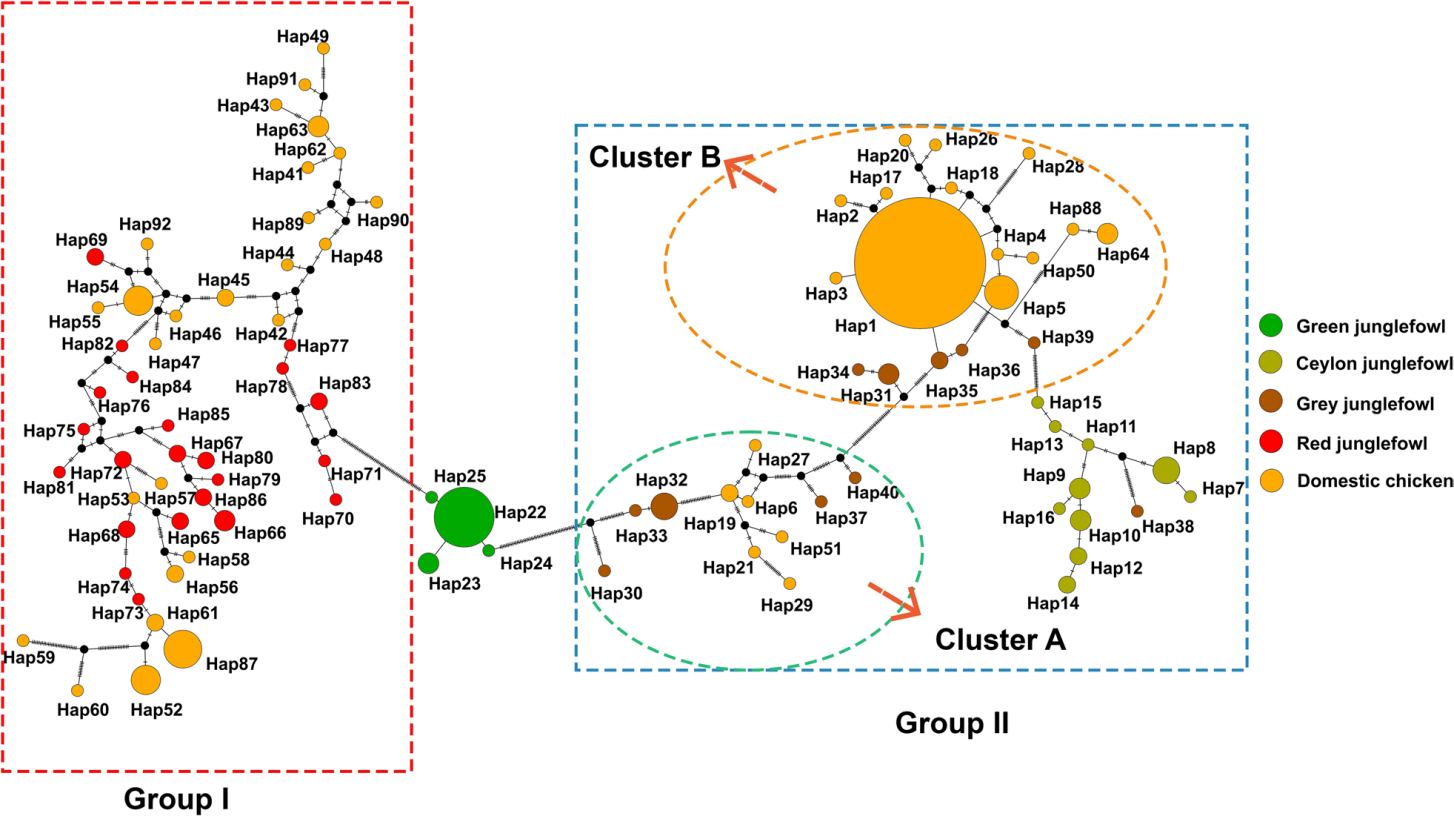
Haplotype network constructed with the *BCO2* gene region. Two different cluster group including group I and group II were indicated by red and blue frames, respectively. And further haplotype clustering part of domestic chickens and grey junglefowl were indicated by green and yellow circles, respectively

To further explore the origin of the yellow skin phenotype in domestic chickens, we constructed a haplotype network using the *BCO2* gene region. Our results show that the entire haplotype network can be divided into two parts including group I and group II, with the green junglefowl haplotype as the dividing point, highlighted by red and blue frames, respectively. The group I consists of the red junglefowl and some domestic chickens, while the group II consists of the grey junglefowl, Ceylon junglefowl, and the remaining domestic chickens (Fig. 6). The clustering results and pattern of the haplotype network were consistent with that observed in the ML tree (Fig. 5b).

Additionally, we found that the haplotype clustering of domestic chickens and grey junglefowl can be further divided into two parts including cluster A and cluster B, highlighted by green and yellow circles, respectively. In either part, it is evident that these domestic chickens’ haplotypes are closer to the grey junglefowl, supporting the yellow skin phenotype in domestic chickens actually originated from the grey junglefowl. Furthermore, we observed that in these two distinct clusters, cluster A is closer to the green junglefowl, while cluster B is relatively more distant. Moreover, we clearly observed Hap1 in the cluster B as being the main haplotype in the introgressed domestic chickens.

## Discussion

Using genomic data from 4 wild junglefowl species and 13 indigenous chicken populations from China, India, and Canada, we systematically investigated the introgression events from the grey junglefowl into domestic chickens, and uncovered additional genomic introgression regions in the chicken genome beyond *BCO2*, providing valuable insights into the contribution of grey junglefowl to the genetic diversity of domestic chickens.

Our phylogenetic and population structure analysis results support a closer genetic relationship between the red junglefowl and domestic chickens. Additionally, we observed in PCA and the phylogenetic tree that the grey junglefowl and Ceylon junglefowl are genetically closer, and admixture analysis further indicates a shared ancestral component within these two junglefowl species. Therefore, our results suggest a sister-group relationship between the grey junglefowl and Ceylon junglefowl. These findings are consistent with the previous analyses based on whole-genome data [6, 7, 16].

Moreover, we confirmed the introgression events from the grey junglefowl into domestic chickens by *D*-statistics analysis. We detected certain genes related to growth regulation, including *IGFBP2* and *TKT*. *IGFBP2* is a member of the *IGFBP* family and further influences the growth and development of animals by regulating the biological activity of *IGF1* [32]. Researches by Tapanainen et al. [33] and Solomon et al. [34] found that overexpression of *IGFBP2* results in fetal growth restriction and leads to a significant decrease in mouse body weight. In domestic chickens, previous studies also indicated its significance as a candidate gene affecting chicken growth traits [32, 35, 36]. *TKT* is an essential enzyme in the pentose phosphate pathway (PPP), and is involved in growth regulation. Existing research has demonstrated that a deficiency of *TKT* could lead to short stature and development delay [37]. In this study, we utilized genomic data to confirm the introgression events at the *IGFBP2* and *TKT* gene region from the grey junglefowl into domestic chickens. However, the functional consequences of this introgression of growth-related genes remain unclear. Therefore, further research and investigation are necessary to uncover the phenotypic differences between the introgressed individuals and the non-introgressed domestic chickens. This will contribute to better understanding the consequences of genetic introgression.

Notably, we simultaneously identified a significant introgression signal (chr24: 6,140,068–6,160,788) from the grey junglefowl into multiple chicken populations, which includes the *BCO2* gene. The *BCO2* gene plays a crucial role in the degradation of skin carotenoids [38]. Multiple studies have confirmed that this gene is a key regulator of the yellow skin phenotype in domestic chickens [5, 38–41]. Taking market demand into consideration, we believe that the phenomenon identified in this study, where the *BCO2* introgression event was found in multiple domestic chicken breeds, may be attributed to the preference for yellow-skinned domestic chickens among people. This preference has likely led to intense selection on this region, ultimately resulting in its retention. Furthermore, we delved into the detail of yellow skin introgression event in domestic chickens by constructing haplotype networks. From the haplotype networks, we observed two distinct clusters include domestic chickens and grey junglefowl. We believe this may reflect the different introgression sources at *BCO2* gene region. In these two distinct clusters, cluster A is closer to the green junglefowl, while cluster B is relatively more distant (Fig. 6). Therefore, we suggest that this may indicate a difference of early and recent introgression event, and possibly reflect multiple genetic flow events between the grey junglefowl and domestic chickens. We can also clearly observe that Hap 1 haplotype in cluster B is the predominant haplotype in the introgressed domestic chickens, which may indicate that this haplotype has undergone widespread dissemination and retention within the domestic chicken population. In summary, these results provide a new insight into the yellow skin phenotype introgression events from the grey junglefowl to the domestic chicken.

Additionally, we also identified two members of the small heat shock protein family, including *CRYAB* and *HSPB2*, which have been introgressed in many domestic chickens. They play crucial roles in cellular defense mechanisms when organisms are exposed to high-temperature environments [42] and may be involved in the high-altitude adaptation of animals [43, 44]. These two genes are located within the significantly introgressed region on chromosome 24, downstream of *BCO2*, spanning from 0.07 Mb to 0.08 Mb. In fact, the introgression event of *BCO2* has been detected in multiple chicken populations due to strong selection on the yellow skin phenotype in domestic chickens [45]. Considering the close physical position of these two genes to *BCO2*, we suggest that the introgression events of *CRYAB* and *HSPB2* are likely a result of a hitchhiking effect.

## Conclusion

Based on whole-genome data of wild junglefowl and domestic chickens, we identified introgression events from the grey junglefowl to domestic chickens in regions beyond *BCO2*. These introgression events have the potential to impact the growth performance. Furthermore, by conducting a detailed haplotype analysis of the *BCO2* region, we believe that the yellow skin phenotype likely underwent strong selection and was retained. And we uncovered evidence of multiple genetic flow between the grey junglefowl and domestic chickens. Therefore, our research is of significant importance in understanding the contribution of grey junglefowl to the genetic diversity of domestic chickens.

### Supplementary Information

**Additional file 1:** **Table S1.** Summary of samples included in this study. **Table S2.** Summary of genome sequencing and mapping statistic. **Table S3.** Results of *D* statistics. **Table S4.** Significant introgression region between the grey junglefowl and XJHT local chickens. **Table S5.** Significant introgression region between the grey junglefowl and Canada local chickens. **Table S6.** Significant introgression region between the grey junglefowl and BJ local chickens. **Table S7.** Significant introgression region between the grey junglefowl and GD local chickens. **Table S8.** Significant introgression region between the grey junglefowl and GX local chickens. **Table S9.** Significant introgression region between the grey junglefowl and HLJ local chickens. **Table S10.** Significant introgression region between the grey junglefowl and Hainan local chickens. **Table S11.** Significant introgression region between the grey junglefowl and Jiangsu local chickens. **Table S12.** Significant introgression region between the grey junglefowl and SD local chickens.

**Additional file 2:** **Fig. S1.** Introgression at the *TIMP3* gene region. **a**. Manhattan plot of *D* values between XJHT local chickens and grey junglefowl. The dashed line indicates the significance threshold (top 1% of the distribution of *D* values). **b**. ML tree constructed with the *TIMP3* gene sequence. The introgressed event was highlighted with orange color, the introgressed XJHT individual and the grey junglefowl were highlighted with red and blue color, respectively. **c**. Mean pairwise sequence divergence at the *TIMP3* gene region between the introgressed XJHT local chickens (INDOC) and either grey junglefowl (GyJ) or remaining non-introgressed XJHT local chickens (ODOC), represents by red and blue lines, respectively. The shaded area represents the *TIMP3* gene region.

**Additional file 3: Fig. S2.** Introgression at the *TKT* gene region. **a**. Manhattan plot of *D* values between Canada local chickens and grey junglefowl. The dashed line indicates the significance threshold (top 1% of the distribution of *D* values). **b**. ML tree constructed with the *TKT* gene sequence. The introgressed event was highlighted with orange color, the introgressed Canada individuals and the grey junglefowl were highlighted with red and blue color, respectively. **c**. Mean pairwise sequence divergence at the *TKT* gene region between the introgressed Canada local chickens (INDOC) and either grey junglefowl (GyJ) or remaining non-introgressed Canada local chickens (ODOC), represents by red and blue lines, respectively. The shaded area represents the *TKT* gene region.

**Additional file 4: Fig. S3.** Introgression at the *CRYAB* and *HSPB2* gene region. **a**. Manhattan plot of *D* values between Canada local chickens and grey junglefowl. The dashed line indicates the significance threshold (top 1% of the distribution of *D* values). **b**. ML tree constructed with the *CRYAB* gene sequence. The introgressed event was highlighted with orange color, the introgressed individuals and the grey junglefowl were highlighted with red and blue color, respectively. **c**. ML tree constructed with the *HSPB2* gene sequence. The introgressed event was highlighted with orange color, the introgressed individuals and the grey junglefowl were highlighted with red and blue color, respectively. **d**. Mean pairwise sequence divergence at the *CRYAB* gene region between the introgressed individuals (INDOC) and either grey junglefowl (GyJ) or remaining non-introgressed domestic chickens (ODOC), represents by red and blue lines, respectively. The shaded area represents the *CRYAB* gene region. **e**. Mean pairwise sequence divergence at the *HSPB2* gene region between the introgressed individuals (INDOC) and either grey junglefowl (GyJ) or remaining non-introgressed domestic chickens (ODOC), represents by red and blue lines, respectively. The shaded area represents the *HSPB2* gene region.

## Data Availability

The whole genome resequencing data of Guangxi Dongzhong Dwarf chickens, Hainan Wenchang chickens and 2 Beijing You chickens are available under the NCBI accession numbers of PRJNA871052 and PRJNA724749. The data for the other domestic chickens, green junglefowl, Ceylon junglefowl, grey junglefowl and red junglefowl were obtained from previous studies [6, 7, 15–19].

## Abbreviations

BCO2: Beta-carotene oxygenase 2
CRYAB: Heat shock protein family B
dxy: Mean pairwise sequence divergence
HSPB2: Heat shock protein family B
IGFBP2: Insulin like growth factor binding protein 2
ML: Maximum likelihood
PCA: Principal component analysis
PC1: The first principal component
TKT: Transketolase
