# Supplementary figures and images for: Significant genomic introgression from grey junglefowl (Gallus sonneratii) to domestic chickens (Gallus gallus domesticus)
Source: J Anim Sci Biotechnol. 2024 Apr 1;15:45. doi: 10.1186/s40104-024-01006-7 (PMC10983685; doi:10.1186/s40104-024-01006-7)

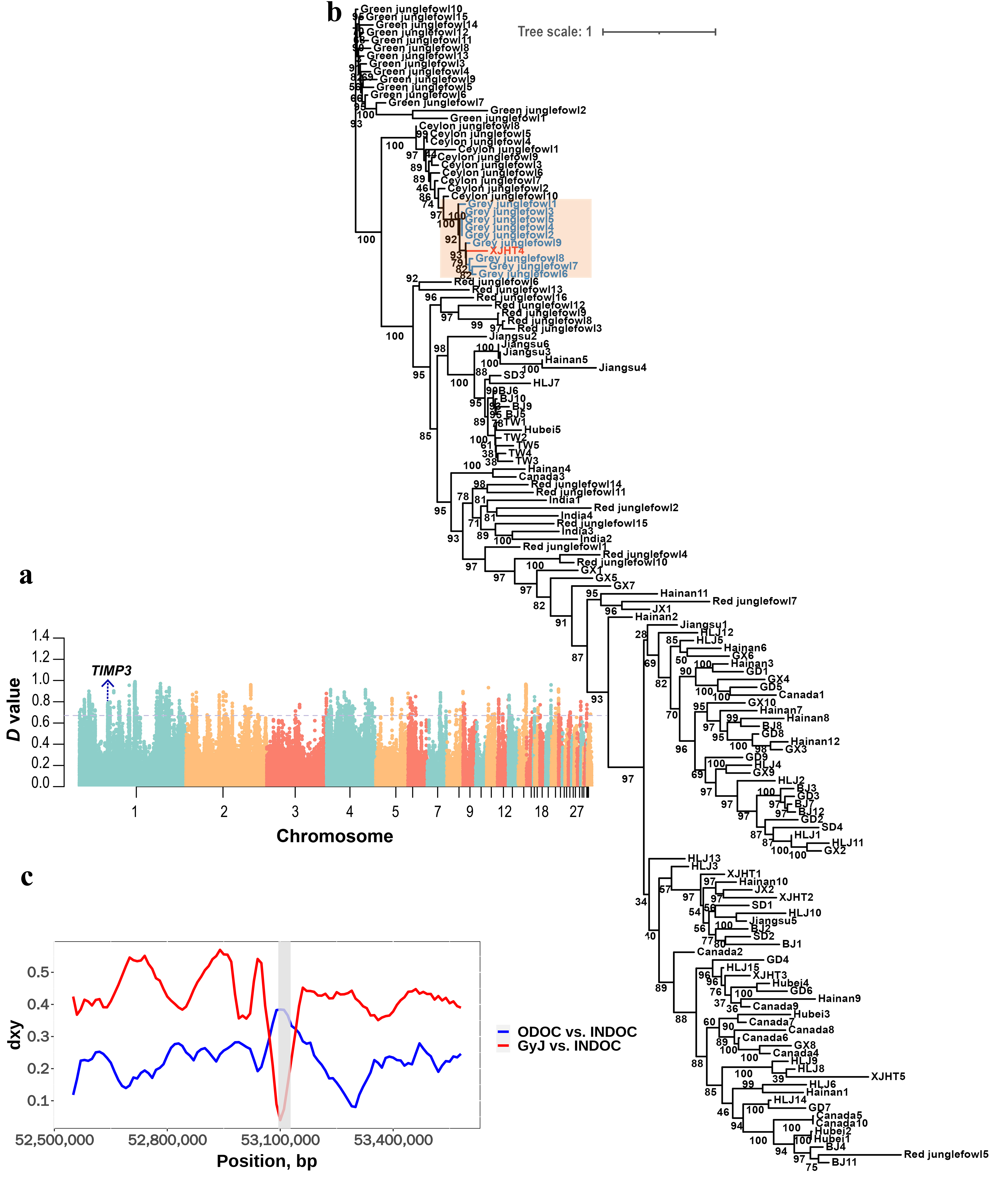

Supplement: Supplementary file 2 — Additional file 2: Fig. S1. Introgression at the TIMP3 gene region. a. Manhattan plot of D values between XJHT local chickens and grey junglefowl. The dashed line indicates the significance threshold (top 1% of the distribution of D values). b. ML tree constructed with the TIMP3 gene sequence. The introgressed event was highlighted with orange color, the introgressed XJHT individual and the grey junglefowl were highlighted with red and blue color, respectively. c. Mean pairwise sequence divergence at the TIMP3 gene region between the introgressed XJHT local chickens (INDOC) and either grey junglefowl (GyJ) or remaining non-introgressed XJHT local chickens (ODOC), represents by red and blue lines, respectively. The shaded area represents the TIMP3 gene region. [file 40104_2024_1006_MOESM2_ESM.tif]

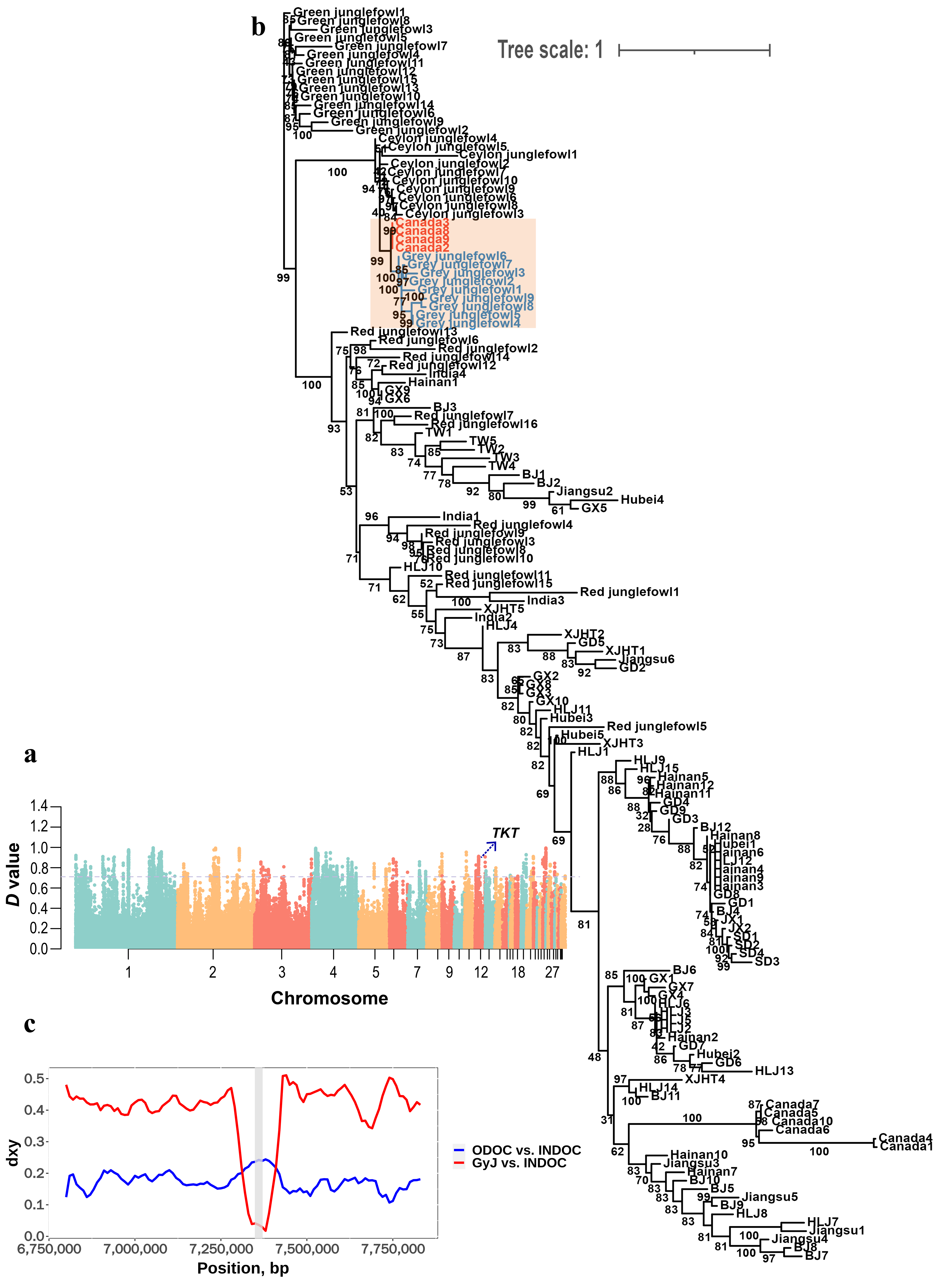

Supplement: Supplementary file 3 — Additional file 3: Fig. S2. Introgression at the TKT gene region. a. Manhattan plot of D values between Canada local chickens and grey junglefowl. The dashed line indicates the significance threshold (top 1% of the distribution of D values). b. ML tree constructed with the TKT gene sequence. The introgressed event was highlighted with orange color, the introgressed Canada individuals and the grey junglefowl were highlighted with red and blue color, respectively. c. Mean pairwise sequence divergence at the TKT gene region between the introgressed Canada local chickens (INDOC) and either grey junglefowl (GyJ) or remaining non-introgressed Canada local chickens (ODOC), represents by red and blue lines, respectively. The shaded area represents the TKT gene region. [file 40104_2024_1006_MOESM3_ESM.tif]

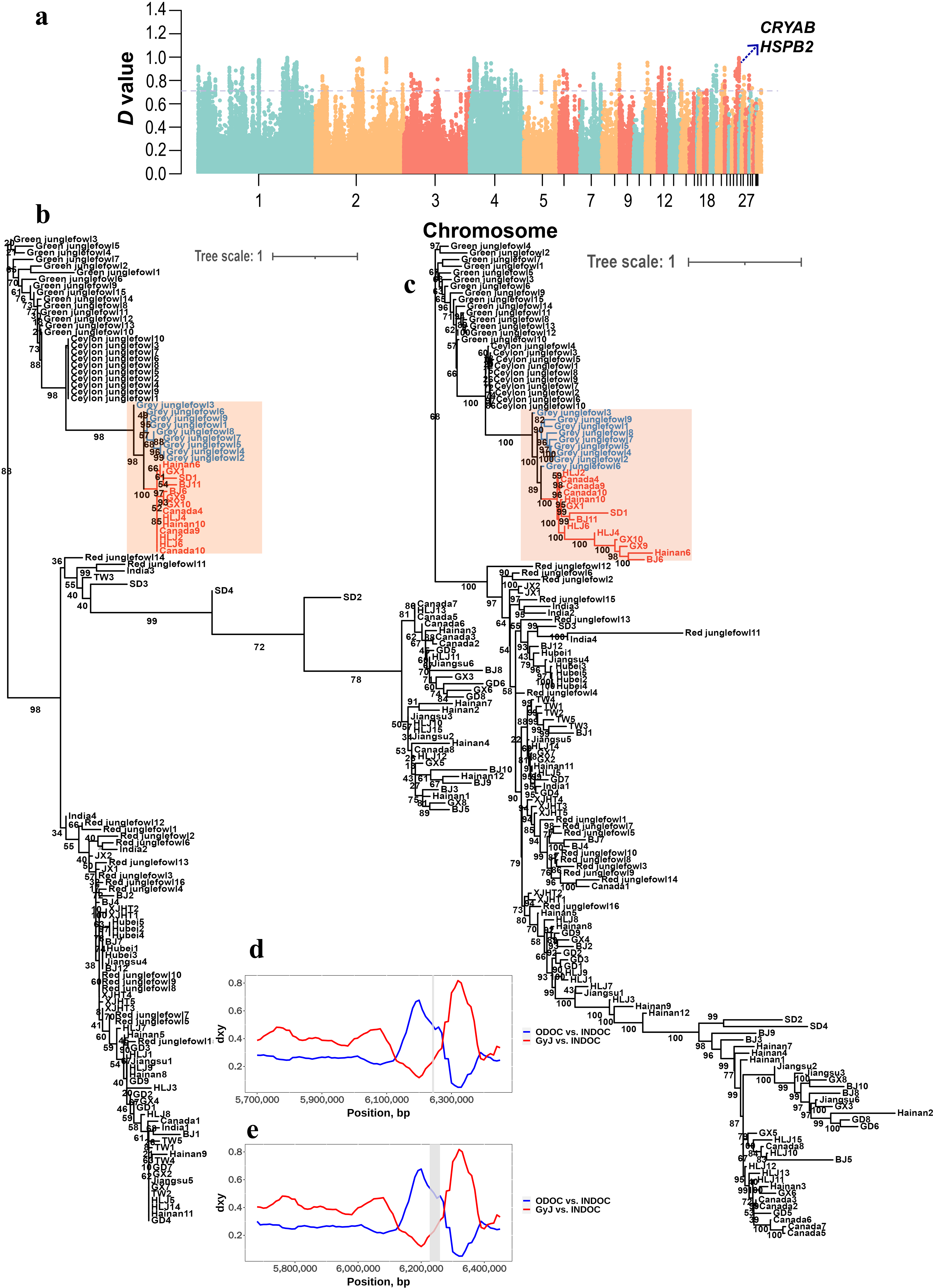

Supplement: Supplementary file 4 — Additional file 4: Fig. S3. Introgression at the CRYAB and HSPB2 gene region. a. Manhattan plot of D values between Canada local chickens and grey junglefowl. The dashed line indicates the significance threshold (top 1% of the distribution of D values). b. ML tree constructed with the CRYAB gene sequence. The introgressed event was highlighted with orange color, the introgressed individuals and the grey junglefowl were highlighted with red and blue color, respectively. c. ML tree constructed with the HSPB2 gene sequence. The introgressed event was highlighted with orange color, the introgressed individuals and the grey junglefowl were highlighted with red and blue color, respectively. d. Mean pairwise sequence divergence at the CRYAB gene region between the introgressed individuals (INDOC) and either grey junglefowl (GyJ) or remaining non-introgressed domestic chickens (ODOC), represents by red and blue lines, respectively. The shaded area represents the CRYAB gene region. e. Mean pairwise sequence divergence at the HSPB2 gene region between the introgressed individuals (INDOC) and either grey junglefowl (GyJ) or remaining non-introgressed domestic chickens (ODOC), represents by red and blue lines, respectively. The shaded area represents the HSPB2 gene region. [file 40104_2024_1006_MOESM4_ESM.tif]
